# Supplementary material for: RRP7A links primary microcephaly to dysfunction of ribosome biogenesis, resorption of primary cilia, and neurogenesis
Source: Nat Commun. 2020 Nov 16;11:5816. doi: 10.1038/s41467-020-19658-0 (PMC7670429; doi:10.1038/s41467-020-19658-0)
Supplement: Supplementary file 1 — Supplementary Information [file 41467_2020_19658_MOESM1_ESM.pdf]

Supplementary information

RRP7A links primary microcephaly to dysfunction of ribosome biogenesis, resorption of primary cilia and neurogenesis

Farooq et al.

Supplementary Figure 1

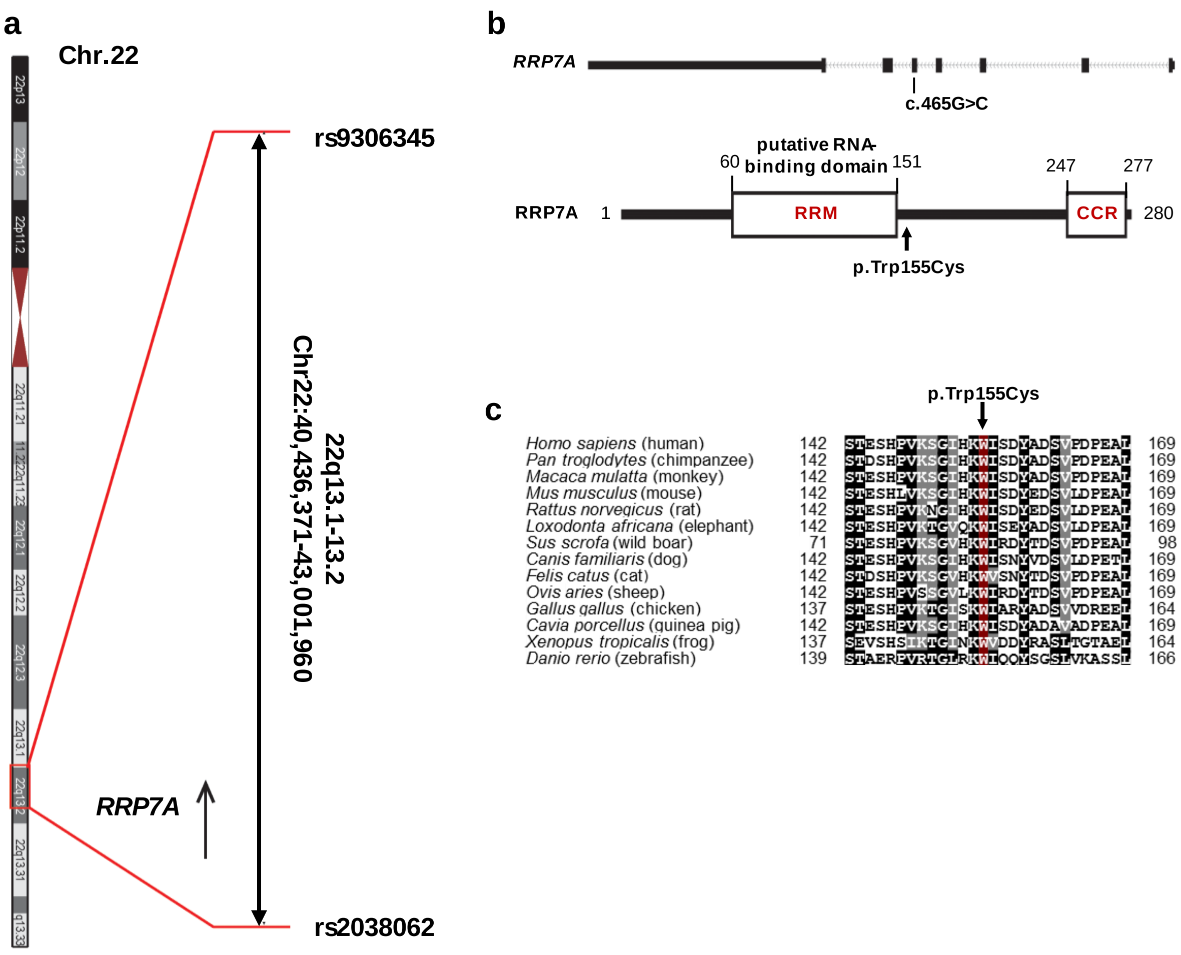

Supplementary Figure 1: Identification of mutation in the *RRP7A* gene.
a, Position of homozygous chromosomal region q13.1-13.2 at chromosome 22 marked by rs9306345 and rs2038062 (physical position 40,436,371-43,001,960, hg38). **b,** Multiple amino acid sequence alignment of RRP7A showing that W155 is highly conserved between different species. **c,** Schematic representation of the *RRP7A* gene (upper panel) showing site of mutation in exon 5. RRP7A protein domain prediction (lower panel) using SMART online tool predicted a RNA recognition motif (RRM; 60-151 amino acid), and a coiled coil region (CCR; 247-277 amino acid). The mutation p.W155C lies just outside the putative RNA binding domain (RRM). CCR: coiled-coiled region.

Supplementary Figure 2

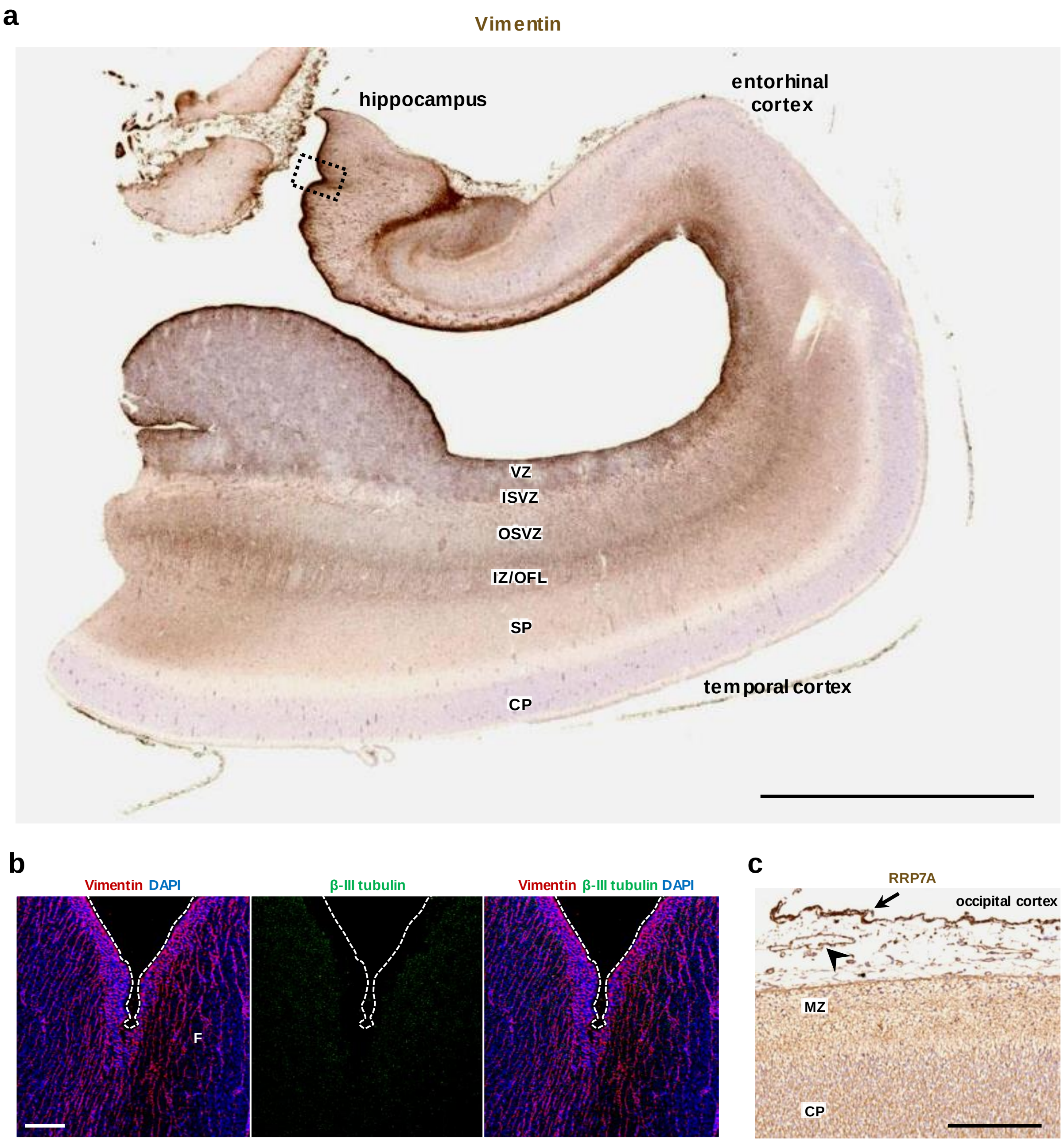

Supplementary Figure 2: Expression of RRP7A in the human midgestation foetal brain aged 19 wpc.
a, DAB staining of the entorhinal cortex and hippocampus for Vimentin. Scale bar, 5 mm. **b**, IFM analysis of the boxed area depicted in (a) showing lack of neurons (βIII-tubulin; *green*) at the outer surface along the hippocampus enriched in RGCs (Vimentin; *red*). Nuclei are stained with DAPI (*blue*). Scale bar, 0.1 mm. **c**, DAB staining of the occipital cortex depicting RRP7A localization to the meninges (arrow) and endothelial cells (arrow head). Scale bar, 0.2 mm. Abbreviations: VZ: ventricular zone, ISVZ: inner subventricular zone, OSVZ: outer subventricular zone, IZ/OFL: intermediate zone/outer fibre layer, SP: subplate, CP: cortical plate, F: Fimbria, MZ: marginal zone.

Supplementary Figure 3

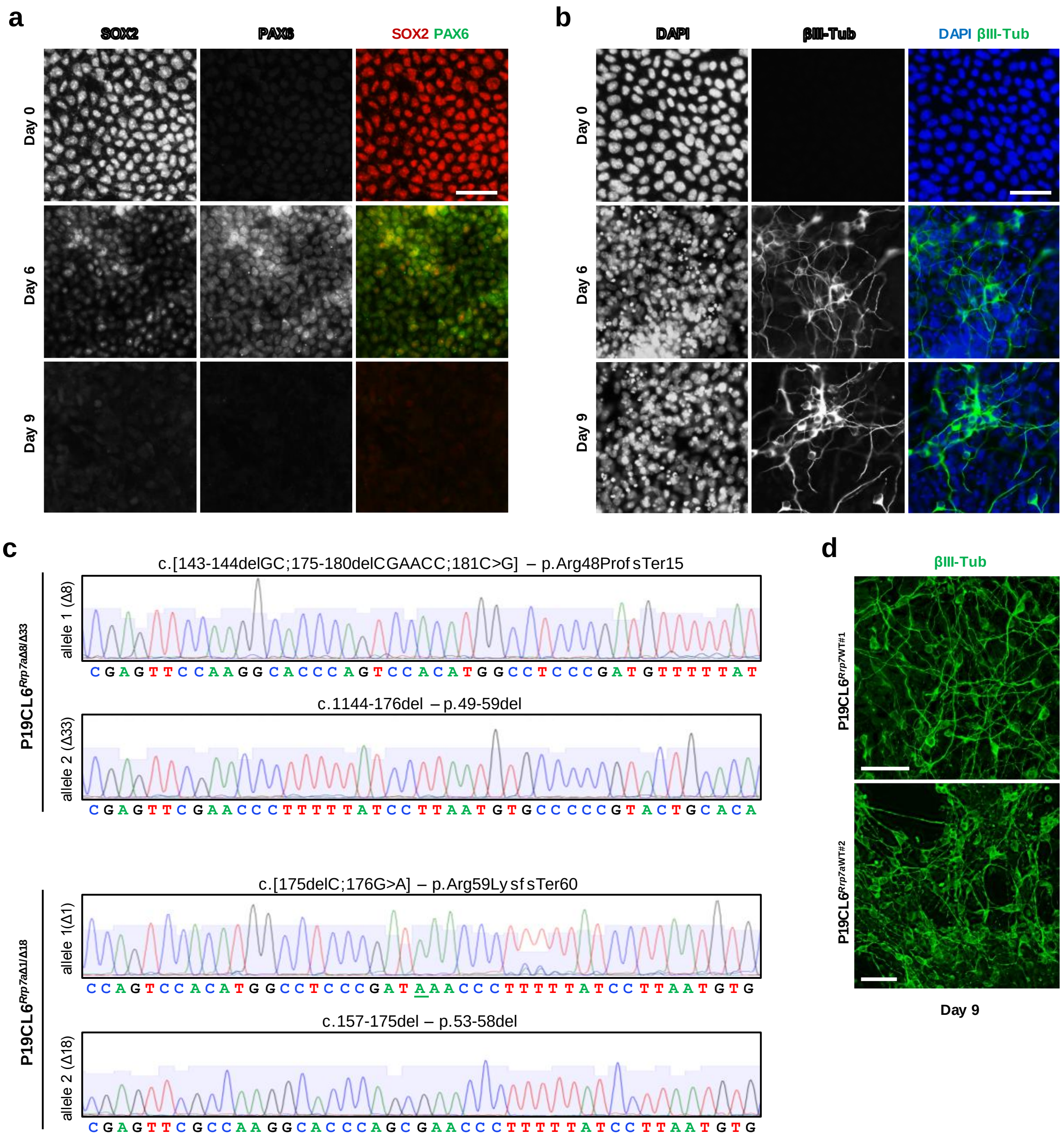

Supplementary Figure 3: *In vitro* neurogenesis and Sanger sequencing of CRISPR clones in P19CL6 cells.
a, IFM analysis of P19CL6 at days 0 (control), 3, 6, and 9 days of retinoic acid (RA) treatment, showing expression of the stem cell marker SOX2 (*red*) and the neurogenesis-controlling factor PAX6 (*green*). Scale bar, 40 mm. **b**, IFM analysis of P19CL6 cells at equivalent days of RA treatment, showing expression of ßIII-tubulin (*green*). Nuclei are stained with DAPI (*blue*). Scale bar, 40 mm. **c**, Sanger sequencing of mutant clones P19CL6$^{Rrp7a\Delta8/\Delta33}$ and P19CL6$^{Rrp7a\Delta1/\Delta18}$. Nucleotides were numbered according to the distance from the 5' end of the sequencing primer and the clones were named according to the total number of nucleotides deleted, which in the case of Δ8 consisted of three microdeletions. Clone Δ1 had a substitution next to the deleted nucleotide, as indicated. The nucleotides flanking the deletions are numbered above diagrams. **d**, IFM analysis showing that WT CRISPR clones form neurons within 9 days of RA treatment ßIII-tubulin (*green*). Scale bar, 40 µm.

Supplementary Figure 4

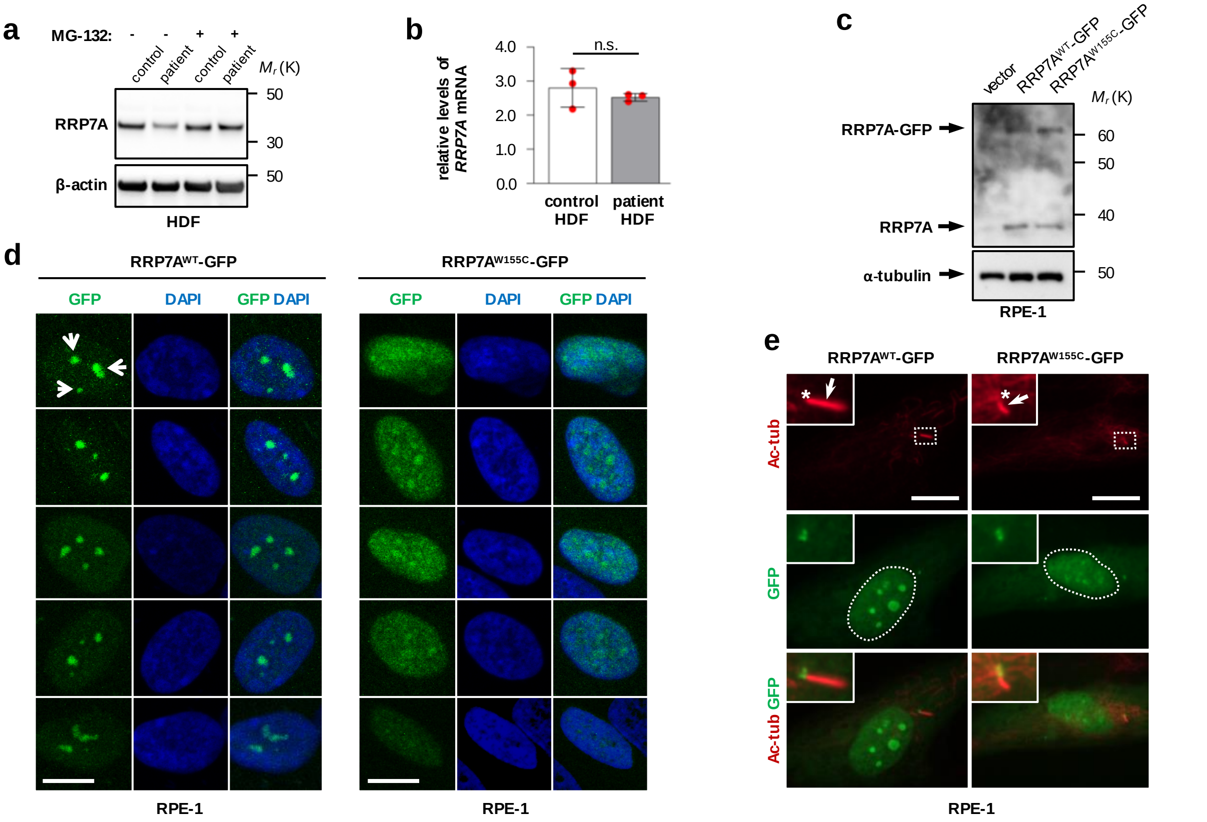

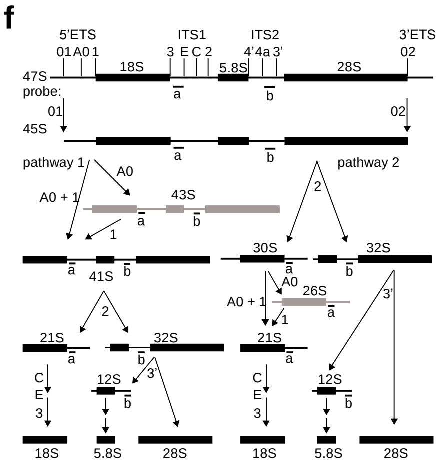

Supplementary Figure 4: Expression and localization of RRP7A and RRP7A constructs in HDFs and hTERT RPE-1 cells.
a, WB analysis of RRP7A expression in control and patient HDFs in the absence and in the presence of the proteasome inhibitor MG-132. β-actin: loading control. **b**, Quantification of relative levels of RRP7A mRNA detected by qRT-PCR in control and patient HDFs (data average of n = 3 independent experiments; *P*=0.454). Data are represented as mean ± SD and significance was determined using an unpaired, two-tailed Student's *t* test. n.s.: not significant. **c**, WB analysis of hTERT RPE-1 (RPE-1) cells transiently transfected with empty vector or vectors expressing GFP-tagged constructs of either WT (RRP7A^{WT}-GFP) or patient mutant (RRP7A^{W155C}-GFP) versions of RRP7A. α-tubulin: loading control. **d**, IFM analysis of localization of RRP7A^{WT}-GFP or RRP7A^{W155C}-GFP (GFP, *green*) to nucleoli (arrow heads) in serum supplemented cultures of RPE-1 cells. Nuclei are stained with DAPI (*blue*). Scale bar 10 µm. **e**, IFM analysis of localization of RRP7A^{WT}-GFP or RRP7A^{W155C}-GFP (GFP, *green*) to nucleoli and primary cilia (closed arrows, Acetylated α-tubulin, Ac-tub, *red*) in serum-depleted cultures of RPE-1 cells. Asterisks indicate the ciliary base and the dotted lines outline the nucleus. Scale bar, 10 µm. **f**, Map of the human pre-rRNA transcript with annotated processing sites and a simplified outline of the two main processing pathways with short-lived precursors in grey. In pathway 1, cleavage of 45S occurs at sites A0 and 1 to produce 41S that is subsequently split into two parts by cleavage at site 2 to yield the direct precursors for the small and large subunit rRNAs, respectively. In pathway 2, this order is reversed, such that 45S is first cleaved at site 2 yielding precursors for the small and large subunit rRNAs. The target sites of oligo nucleotide probes a and b are indicated on the map.

Supplementary Figure 5

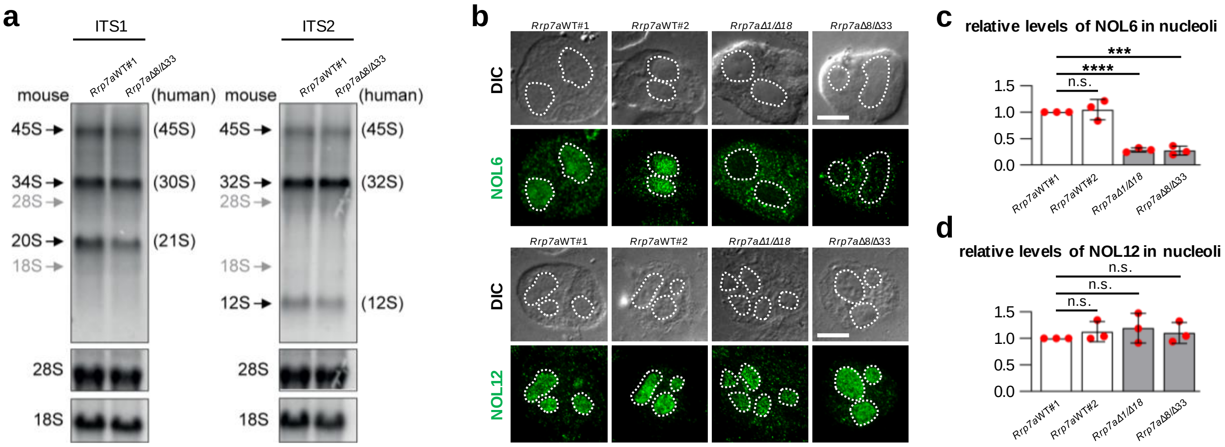

Supplementary Figure 5: rRNA processing and nucleolar NOL6 localization are disrupted in P19CL6 mutant clones.

a, Northern blots of parallel gel runs of total RNA samples from P19CL6$^{\mathit{Rrp7a}\mathrm{WT\#1}}$ and mutant clone P19CL6$^{\mathit{Rrp7a}\Delta 8/\Delta 33}$. Black arrows indicate processing intermediates inferred from the analyses and grey arrows mark the migration of mature rRNA species as inferred from re-probing of the filters with probes targeting these RNA species. Hybridization against 45S, and mature 18S and 28S rRNA were used as internal molecular markers. **b,** IFM analysis on the localization of NOL6 and NOL12 (*green*) to nucleoli (marked by stippled lines by differential interference contrast microscopy, DIC) in cultures of WT and mutant clones. Scale bar, 5 µm. **c,** Quantification of the relative levels of NOL6 in nucleoli in WT and mutant clones shown in (b) ($^{\mathit{Rrp7a}\mathrm{WT\#1}}$ = 98 cells, $^{\mathit{Rrp7a}\mathrm{WT\#2}}$ =101 cells, $^{\mathit{Rrp7a}\Delta 1/\Delta 18}$ = 95 cells, $^{\mathit{Rrp7a}\Delta 8/\Delta 33}$ = 94 cells, data average of n = 3 independent experiments; $^{\mathit{Rrp7a}\mathrm{WT\#2}}$: *P*=0.688, $^{\mathit{Rrp7a}\Delta 1/\Delta 18}$: *P*=7.296E-06, $^{\mathit{Rrp7a}\Delta 8/\Delta 33}$: *P*=1.227E-04). **d,** Quantification of the relative levels of NOL12 in nucleoli in WT and mutant clones shown in (b) ($^{\mathit{Rrp7a}\mathrm{WT\#1}}$ = 84 cells, $^{\mathit{Rrp7a}\mathrm{WT\#2}}$ = 92 cells, $^{\mathit{Rrp7a}\Delta 1/\Delta 18}$ = 90 cells, $^{\mathit{Rrp7a}\Delta 8/\Delta 33}$ = 87 cells, data average of n = 3 independent experiments. $^{\mathit{Rrp7a}\mathrm{WT\#2}}$: *P*=0.305, $^{\mathit{Rrp7a}\Delta 1/\Delta 18}$: *P*=0.296, $^{\mathit{Rrp7a}\Delta 8/\Delta 33}$: *P*=0.408). Data are represented as mean ± SD and significance was determined using a two-tailed Student's *t* test. ****P<0.0001, n.s.: not significant.

Supplementary Figure 6

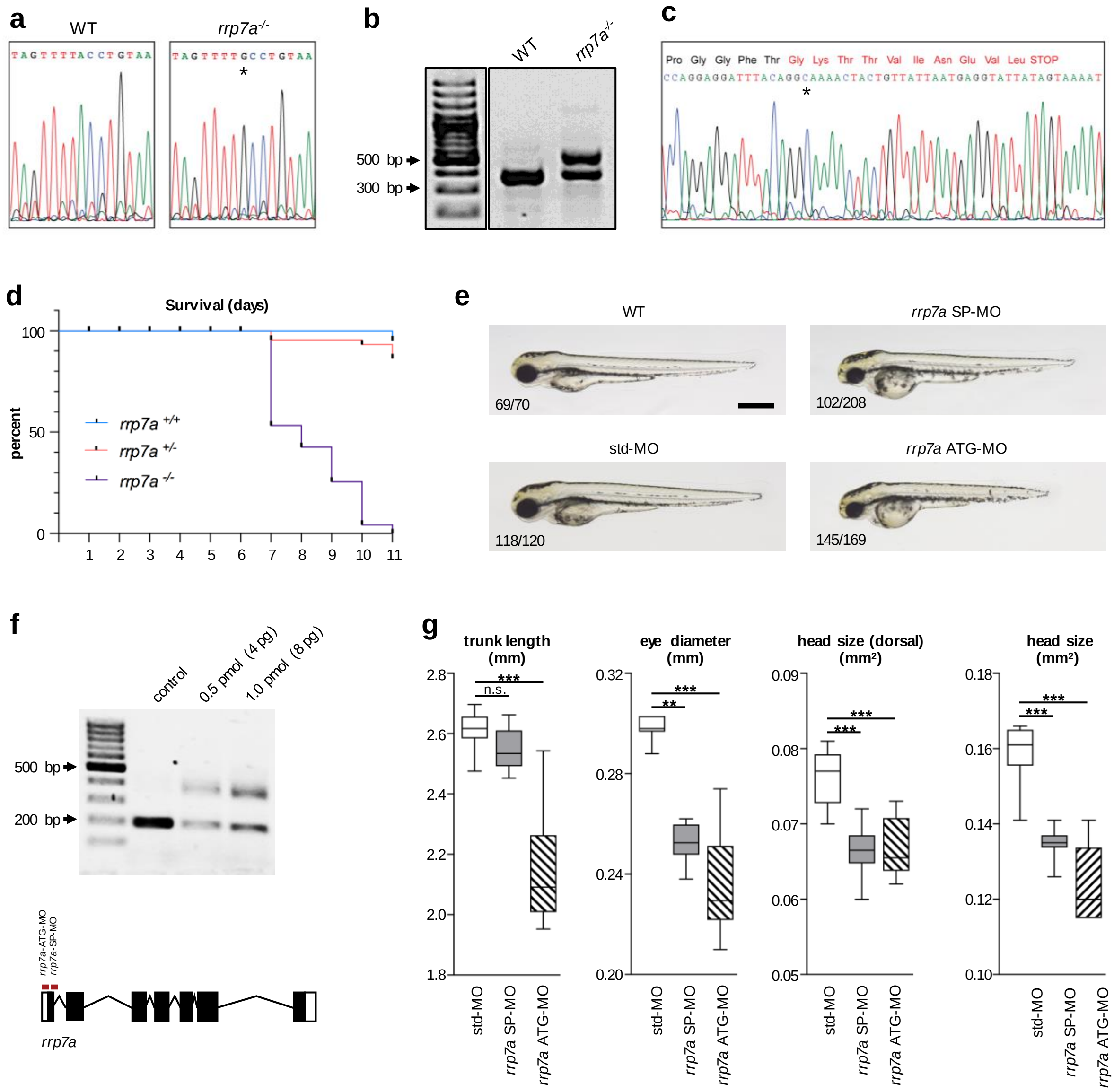

Supplementary Figure 6: Zebrafish *rrp7a* mutants and morphants.

a, Reverse-complement *rrp7a* DNA sequence of WT and homozygous mutant larvae. Mutants carry a point mutation in the donor-splice site of exon 1 in *rrp7a* (ivs1 +2 T>C, NM_001017579), leading to aberrant splicing of *rrp7a* mRNA, with inclusion of 191 bp of intronic sequence. The inclusion of intronic sequence leads to a shift in the reading frame, inclusion of 10 abnormal amino acid residues at position 18-27 followed by a premature stop codon. The deduced mutant protein is truncated C-terminal to the RRM and contains 27 amino acid residues in total. The ivs1 + 2 T>C mutation is marked with *. **b,** RT-PCR fragments from *rrp7a* mRNA extracted from WT and homozygous mutant larvae. **c,** DNA sequence of aberrantly spliced *rrp7a* mRNA (upper right band in B). Ivs1 + 2 T>C mutation is marked with an asterisk. Abnormal amino residues are shown in red. **d**, Kaplan-Meier survival curves of *rrp7*$^{+/+}$ (light blue line) (n=22, biologically independent animals), *rrp7*$^{+/-}$ (red line) (n=45, biologically independent animals), and *rrp7*$^{-/-}$ (purple line) (n=47, biologically independent animals). **e**, Phenotypes of *rrp7a* morphants. Embryos were injected with standard control morpholino (std-MO), splice blocking morpholino (SP-MO) and translation blocking morpholino (ATG-MO). scale bar, 0.5 mm. **f**, upper: RT-PCR showing aberrant splicing in embryos injected with SP-MO. lower: target position of ATG-MO and SP-MO in *rrp7a* mRNA. **g**, quantification of trunk length, eye diameter, dorsal head size and lateral head size (n=10, biologically independent experiments) were plotted as box and whiskers, whiskers min to max; significance was determined using a one-way ANOVA on ranks. ***P<0.001, **P=0.006, n.s.: not significant (P=0.384).

Supplementary Figure 7

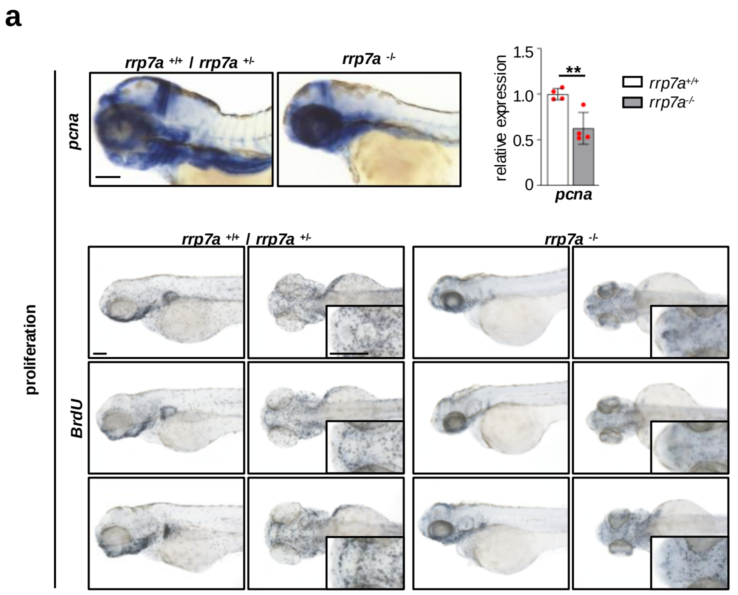

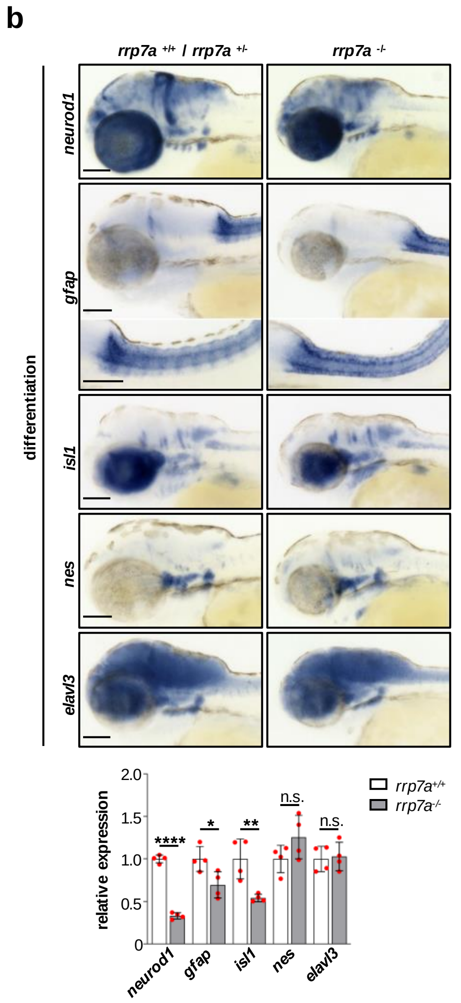

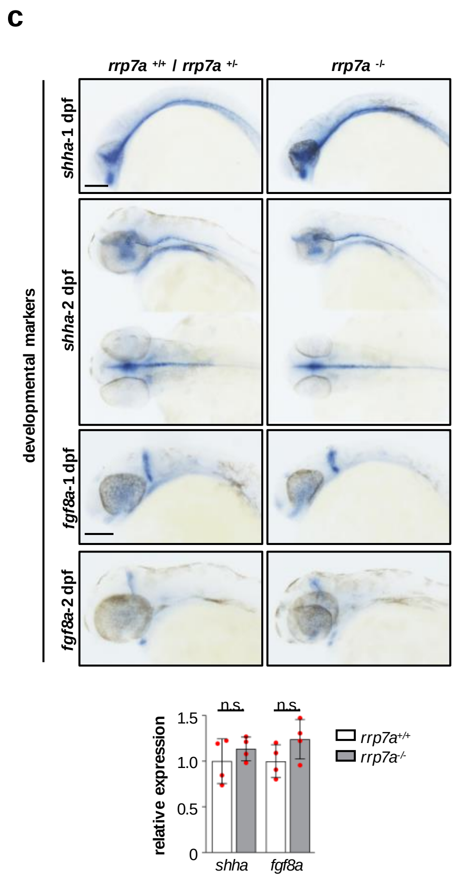

Supplementary Figure 7: Analysis of proliferation, differentiation and developmental markers in zebrafish embryos. a, proliferation analysis. Upper panel: mRNA expression analysis of proliferation marker *pcna* by *in situ* hybridization (left) and qRT-PCR (right) (*P*=0.007). Panel 2-4: BrdU labelling of *rrp7*$^{+/+}$ / *rrp7*$^{+/-}$ (left) and *rrp7*$^{-/-}$ larvae (right). **b**, differentiation analysis. mRNA expression analysis of neural differentiation markers *neurod1* (*P*=4.842E-07), *gfap* (*P*=0.028), *isl1* (*P*=0.008), *nes* (*P*=0.141) and *elavl3* (*P*=0.812) by *in situ* hybridization (panel 1-5) and qRT-PCR (lower panel). **c**, mRNA expression analysis of developmental markers *shha* (*P*=0.365)and *fgf8a* (*P*=0.138) by *in situ* hybridization (panel 1-4) and qRT-PCR (lower panel). All assays have been performed on 3 dpf larvae unless otherwise mentioned. Scale bars, 0.1 mm. All quantifications present average of n = 4 biologically independent experiments. Data are represented as mean ± SD and significance was determined using a two-tailed Student's *t* test (n=4). ***P<0.001, **P<0.01, *P<0.05, n.s.: not significant.

Supplementary Figure 8

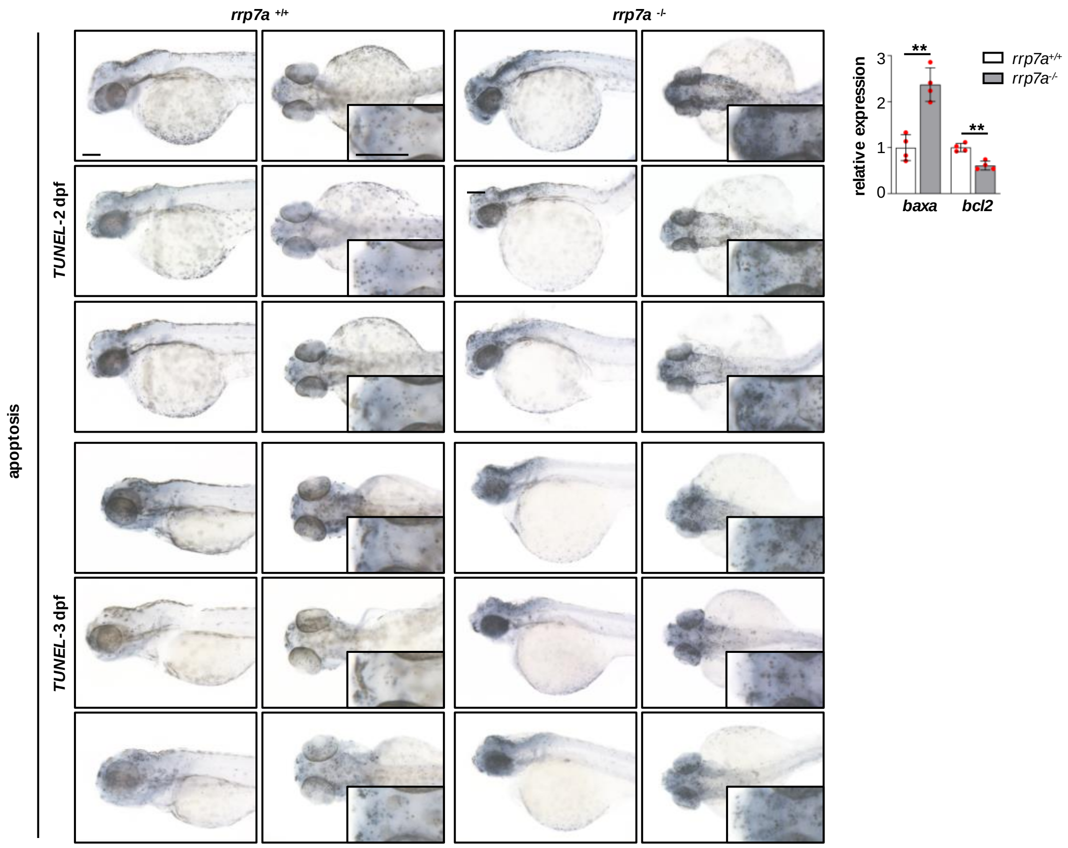

Supplementary Figure 8: Analysis of apoptosis in zebrafish embryos. Left: results of TUNEL apoptosis assay in 2 dpf and 3 dpf zebrafish. Scale bars, 0.1 mm. Right: qRT-PCR analysis of apoptosis markers, *baxa* (*P*=0.001) and *bcl2* (*P*=0.001), in 3 dpf larvae. Data are represented as mean ± SD and significance was determined using an unpaired, two-tailed Student's *t* test (n=4). ***P*<0.01.

Supplementary Figure 9

a

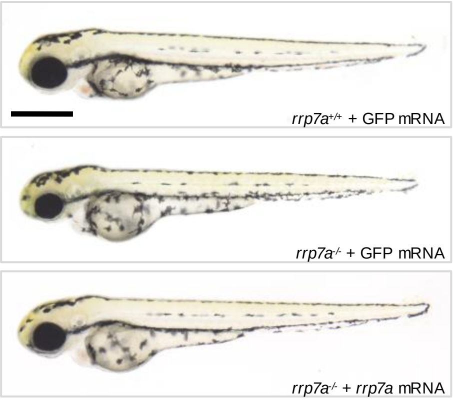

b

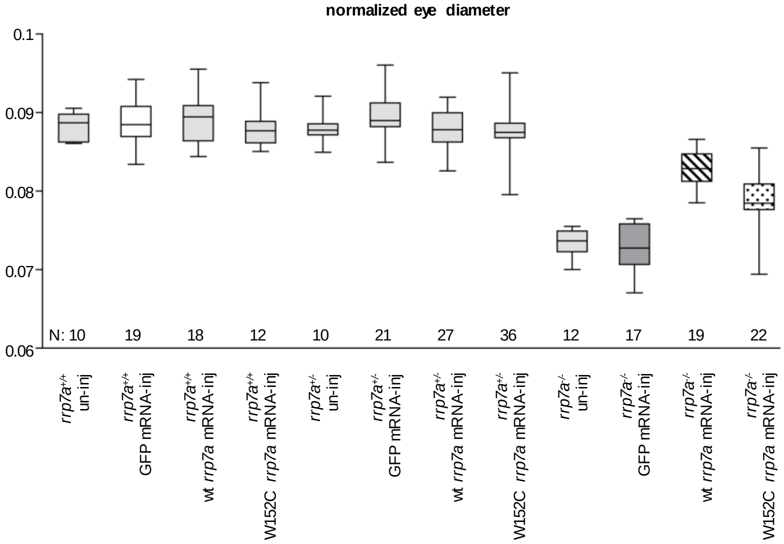

Supplementary Figure 9: Rescue of zebrafish embryos. a, representative images of 3 dpf *rrp7*$^{+/+}$ larvae injected with GFP mRNA (upper) and *rrp7*$^{-/-}$ injected with GFP mRNA (middle) and *rrp7a* mRNA (lower). Scale bar is 0.5 mm. **b**, normalized eye diameter quantification of 3 dpf larvae (*rrp7*$^{+/+}$, *rrp7*$^{+/-}$ and *rrp7a*$^{-/-}$), including the groups presented in Fig. 5j, were plotted as box and whiskers, whiskers min to max. The embryos were un-injected or injected with GFP mRNA, wt *rrp7a* mRNA or mutant *rrp7a* (p.W152C) mRNA.

Supplementary Table 1

Patient	Age	Sex	HC (cm), SD	Intellectual disability	Speech impairment
IV.3	30	Male	44, -8 SD	Moderate	NO
V.4	15	Female	45, -7 SD	Mild	NO
V.6	12	Female	42.5, -8 SD	Mild	YES
V.8	8	Female	41, -7 SD	Moderate	NO
V.10	10	Female	42.5, -6 SD	Mild	NO
V.12	27	Male	47, -6 SD	Moderate	YES
V.14	14	Female	45.5, -7 SD	Severe	YES
V.15	11	Female	43.5, -6 SD	Mild	YES

Supplementary Table 1: Clinical phenotypes of affected individuals.
Clinical characteristics of eight effected family members. HC: Head Circumference SD: Standard deviations from age-adjusted mean.

Supplementary Table 2

Primary antibody	Species	Supplier	Dilution
Acetylated α-tubulin	Mouse	Sigma-Aldrich, T7451	1:2000 (IFM)
ARL13B	Rabbit	Proteintech, 17711-1-AP	1:600 (IFM)
α-tubulin	Mouse	Sigma-Aldrich, T5168	1:10,000 (WB)
βIII-tubulin	Rabbit	AbCam, AB18207	1:200 (WB)<br>1:2000 (IFM, DAB)
BrdU	Rat	CiteAb, MA1-82718	1:300
GFP	Rabbit	Santa Cruz, sc-8334	1:500 (WB)
GFAP	Rabbit	DAKO, Z0334	1:1000 (DAB)
MAP2	Rabbit	Abcam, AB32454	1:2000 (WB)
MAP2	Chicken	Abcam, AB5392	1:1000 (IFM)
DCTN1	Mouse	BD Bioscience, 610474	1:1000 (WB)<br>1:500 (IFM)
NOL6	Rabbit	Thermo Fisher Scientific, PA5-30807	1:1000 (WB)
NOL6	Rabbit	Sigma-Aldrich, HPA055891	1:100 (IFM)
NOL12	Rabbit	Abcam, AB111704	1:1000 (WB)<br>1:200 (IFM)
PAX6	Rabbit	Abcam, AB5790	1:300 (WB)<br>1:300 (IFM)
Pericentrin-2	Goat	Santa Cruz, sc-28145	1:300 (IFM)
phospho-RB	Rabbit	Cell Signaling, 9308s	1:300 (WB)<br>1:500 (IFM)
phospho-CDK1^{T161}	Rabbit	Cell Signaling, 9114	1:200 (WB)
RRP7A	Rabbit	AbCam, AB185225	1:2000 (WB)
RRP7A	Rabbit	Sigma, HPA001586	1:500 (IFM, DAB)
RRP7A	Mouse	Santa Cruz, SC377210	1:500 (IFM)
SOX2	Mouse	R&D system, MAB2018	1:400 (IFM)
Vimentin	Chicken	Thermo Fischer, PA1-16759	1:200 (IFM)
Vimentin	Mouse	Dako, M072501-2	1:100 (DAB)

Supplementary Table 2: List of primary antibodies.
Abbreviations: WB: western blotting, IFM: immunofluorescence microscopy, DAB: 3,3'-diaminobenzidine.

Supplementary Table 3

Secondary antibody	Conjugation	Supplier	Dilution
IFM/DAB			
donkey-anti-mouse IgG	Alexa Flour 488	Invitrogen A-21202	1:600
donkey-anti-mouse IgG	Alexa Flour 568	Invitrogen A-10037	1:600
donkey-anti-rabbit IgG	Alexa Flour 488	Invitrogen A2106	1:600
donkey-anti-rabbit IgG	Alexa Flour 568	Invitrogen A10042	1:600
donkey-anti-goat IgG	Alexa Flour 568	Invitrogen A-11057	1:600
goat-anti-chicken IgG	Alexa Flour 568	Invitrogen A11041	1:600
goat-anti-chicken IgG	Alexa Flour 647	Invitrogen A31571	1:600
WB			
pig anti rabbit IgG	HRP conjugated	DAKO, P0217	1:5000
goat anti mouse IgG	HRP conjugated	DAKO, P0447	1:5000
rabbit anti goat IgG	HRP conjugated	DAKO, P0449	1:5000

Supplementary Table 3: List of secondary antibodies.
Abbreviations: WB: western blotting, IFM: immunofluorescence microscopy, DAB: 3,3'-diaminobenzidine.

Supplementary Table 4

Name	5' – 3' sequence
qRT-PCR primers	
zActb1-RT-F	AGATCTTCACTCCCCTTGTTCAC
zActb1-RT-R	AAACCGGCTTTGCACATACC
zEef1a1-RT-F	CTTTCTGTTACCTGGCAAAGGG
zEef1a1-RT-R	CGTGGCCAATAACCACGATG
zElavl3-RT-F	GTCAGAAAGACATGGAGCAGTTG
zElavl3-RT-R	GAACCGAATGAAACCTACCCC
zFgf8a-RT-F	ATGAACAGGAGGGGGAAACT
zFgf8a-RT-R	GAAAATGCAGTCTTTTCCCAGA
zGfap-RT-F	GGTCCATGAGGAGGAGATGA
zGfap-RT-R	CCATGGCCTCAAACTGAGAT
zIsl1-RT-F	CAAATGGCAGCAGAGCCCAT
zIsl1-RT-R	GGACGCGGGTTGTTTTCTCA
zNestin-RT-F	GAGCGAGATAAACGGTCAGC
zNestin-RT-R	TGTGTTCTGAAAGCACTGGC
zNeurod1-RT-F	CAAGCTTTCAACACACCCTAGA
zNeurod1-RT-R	ACTTGTCGGTCCAGTTCGAG
zPcna-RT-F	GGCAACATCAAGCTCTCACA
zPcna-RT-R	TGCACTGGCTCATTCATCTC
zShha-RT-F	AAGCCCACATTCATTGCTCT
zShha-RT-R	CCTTCTGTCCTCCGTCCTG
ISH probe primers	
zElavl3-F	GCACCATGGAAACTCAGGTG
zElavl3-R	GGAGAATCTGAAGCGCTGGG
zFgf8a-F	TATTTGTGCTGCGCTCCTC
zFgf8a-R	TGCGTTTAGTCCGTCTGTTG
zGfap-F	TCATTCTCCTCCACCATGGAGTC
zGfap-R	AGCGGTCAAGTCTGGCTTAG
zIsl1-F	AGGAGATGAGTTCGCTCTGC
zIsl1-R	GATCCACTTTCGCTGGAGTC
zNestin-F	GGCAACATACAGAGCTTTGCTGG
zNestin-R	CAAGGAAAGATGAAGCCAGTGC
zNeurod1-F	TAACCAGAGCATCCCATCAGC
zNeurod1-R	GCGGCTTGACGTGAAAGATG
zPcna-F	AGTGGACAGCACATCTGCAC
zPcna-R	TGTGACCGTCTTGGACAGAG
zRrp7a-F	GCGTGCGTTATTCCAGGAG
zRrp7a-R	GGCTTGAACTTCCTCTGTGC
zShha-F	TCTCGCATTAAGTGGCTGTG
zShha-R	TGGAGTCTCGGTCTGTGAAC
rrp7a cloning primers	
zRrp7a-cds-F-XbaI	CTGATCTAGAATGGCGCCGTCCGCGAATAA
zRrp7a-cdsR-XbaI	CGCTTCTAGATCAGTAGGGCTTGAACTTCC
zRrp7a-sdm-F	GGCCTGCGCAAGTGCATCCAGCAGTACTC
zRrp7a-sdm-R	GAGTACTGCTGGATGCACTTGCGCAGGCC
Morpholinos	
Rrp7a-ATG-MO	GCGCCATCTTAGTTTACATGCGTCA
Rrp7a-SP-MO	AACAGTAGTTTTACCTGTAAATCCT
Std-MO	CCTCTTACCTCAGTTACAATTTATA
rrp7a genotyping/morpholino test primers	
Rrp7a-gen-F	TTCGGGTGACGCATGTAAAC
Rrp7a-gen-R	ACGAAGAGTGTTCTGTCGGC
Northern blotting analyses of rRNA processing intermediates	
Human ITS1	TGGGTGTGCGGAGGGAAGC
Human ITS2	ACGCCGCCGGGTCTGCGCTTA
Human 18S	CCAGACAAATCGCTCCACCAACTAAG
Human 28S	GCTCCCGTCCACTCTCGAC
Mouse ITS1	ACGCCGCCGCTCCTCCACAGTCTCCCGTT
Mouse ITS2	ACCCACCGCAGCGGGTGACGCGATTGATCG
Mouse 18S	GGCCGTGCGTACTTAGACATGCATG
Mouse 28S	TTACACACTCCTTAGCGGATTCCGAC
Zebrafish 5'ETS (somatic)	GAAGAGAGCTCTTCCTCGCTCACG
Zebrafish ITS1 (somatic)	GGAGATCCCCTCTCGAACCCGT
Zebrafish ITS2 (somatic)	TGACGGTCTGCGCTTAGGAGGACGTA
Zebrafish 18S	TGTGTACAAAGGGCAGGGAC
Zebrafish 28S	ACGTGCAAATCGGTCGTCCGAC
qRT-PCR of mature zebrafish rRNA	
Zebrafish 18S forward	ATGGGCGGATCAACGTGTGC
Zebrafish 18S reverse	TGTGTACAAAGGGCAGGGAC
Zebrafish 28S forward	ACGTGCAAATCGGTCGTCCGAC
Zebrafish 28S reverse	GGTCTGATGAGCGTCGGCATC

Supplementary Table 4: Oligonucleotides used in the study.
