# Supplementary material for: RRP7A links primary microcephaly to dysfunction of ribosome biogenesis, resorption of primary cilia, and neurogenesis
Source: Nat Commun. 2020 Nov 16;11:5816. doi: 10.1038/s41467-020-19658-0 (PMC7670429; doi:10.1038/s41467-020-19658-0)
Supplement: Supplementary file 3 — Reporting Summary [file 41467_2020_19658_MOESM3_ESM.pdf]

nature research

Corresponding author(s): Søren Tvorup Christensen & Lars Allan Larsen

Last updated by author(s): Sep 22, 2020

Reporting Summary

Nature Research wishes to improve the reproducibility of the work that we publish. This form provides structure for consistency and transparency in reporting. For further information on Nature Research policies, see our Editorial Policies and the Editorial Policy Checklist.

Statistics

For all statistical analyses, confirm that the following items are present in the figure legend, table legend, main text, or Methods section.

n/a	Confirmed	
☐	☒	The exact sample size (n) for each experimental group/condition, given as a discrete number and unit of measurement
☐	☒	A statement on whether measurements were taken from distinct samples or whether the same sample was measured repeatedly
☐	☒	The statistical test(s) used AND whether they are one- or two-sided<br>*Only common tests should be described solely by name; describe more complex techniques in the Methods section.*
☐	☒	A description of all covariates tested
☐	☒	A description of any assumptions or corrections, such as tests of normality and adjustment for multiple comparisons
☐	☒	A full description of the statistical parameters including central tendency (e.g. means) or other basic estimates (e.g. regression coefficient) AND variation (e.g. standard deviation) or associated estimates of uncertainty (e.g. confidence intervals)
☐	☒	For null hypothesis testing, the test statistic (e.g. F, t, r) with confidence intervals, effect sizes, degrees of freedom and P value noted<br>*Give P values as exact values whenever suitable.*
☒	☐	For Bayesian analysis, information on the choice of priors and Markov chain Monte Carlo settings
☒	☐	For hierarchical and complex designs, identification of the appropriate level for tests and full reporting of outcomes
☒	☐	Estimates of effect sizes (e.g. Cohen's d, Pearson's r), indicating how they were calculated

Our web collection on statistics for biologists contains articles on many of the points above.

Software and code

Policy information about availability of computer code

Data collection	Immunofluorescence images and z-stacks were acquired using CellSens Dimension software version 1.7 (Olympus). Western blotting Images obtained from the FUSION FX chemiluminescence system were acquired using the Evolution-Capt EDGE software version 18.01 (Vilber Lourmat). Zebrafish images were acquired using ZEN2 (Blue Edition) Imaging Software V1.0 (Carl Zeiss Microscopy GmbH, Göttingen, Germany). qRT-PCR data was collected using Quantstudio v.1.5.0 (Thermo-Fischer).
Data analysis	MRI Images were analysed using syngo fastView (version 1.0.0.3, Siemens AG). Genetic data analysis was performed using Chromosomal Analysis Suite v. 2.1 (Affymetrix), FASTLINK v.1.0 (https://www.ncbi.nlm.nih.gov/CBBresearch/Schaffer/fastlink.html), Peak Scanner 1.0 (Applied Biosystems) and chromaspro v. 2.1 (Technelysium Pty Ltd) software. NGS data was filtered and aligned to the Human Genome reference (hg38) using Burrows Wheel Alignment. Variants were analysed using the Genome Analysis Toolkit. Immunofluorescence intensities and cell confluency were enumerated with CellSens Dimension software version 1.7 (Olympus), and Image J version 2.0 and Adobe Photoshop CS6 version 13.0 were used for image processing. Band intensities in western blotting were analysed using ImageJ version 2.0 and UN-SCAN-IT gel version 6.1 (Silk Scientific Corporation). Northern blot band intensities were analysed using ImageQuant TL version 7.0 (Cytiva). Zebrafish embryos were measured using ImageJ software (v. 2.0.0-rc-69/1.52n). Statistical analyses were performed using GraphPad Prism v. 8.4.3, Sigmaplot 14.0 and Microsoft Excel version 2010.

For manuscripts utilizing custom algorithms or software that are central to the research but not yet described in published literature, software must be made available to editors and reviewers. We strongly encourage code deposition in a community repository (e.g. GitHub). See the Nature Research guidelines for submitting code & software for further information.

Data

Policy information about availability of data

All manuscripts must include a data availability statement. This statement should provide the following information, where applicable:

- Accession codes, unique identifiers, or web links for publicly available datasets
- A list of figures that have associated raw data
- A description of any restrictions on data availability

All relevant data supporting the key findings of this study are available within the article and its Supplementary Information files or from the corresponding author upon request. Source data, including full scan images of blotting data used in this study, are shown in Source Data file, which is provided with this paper. Exome sequencing data from the single individual, which was analyzed in the family, is not available due to concerns over patient privacy. Sequence data from the following GenBank accession numbers were used to generate expression constructs: NM_001017579.1 and NM_015703.4.

Field-specific reporting

Please select the one below that is the best fit for your research. If you are not sure, read the appropriate sections before making your selection.

☒ Life sciences ☐ Behavioural & social sciences ☐ Ecological, evolutionary & environmental sciences

For a reference copy of the document with all sections, see nature.com/documents/nr-reporting-summary-flat.pdf

Life sciences study design

All studies must disclose on these points even when the disclosure is negative.

Sample size	Sample sizes in cell culture and zebrafish studies were chosen to meet requirements for statistical analyses (n=3 or more independent experiments)
Data exclusions	No data were excluded from the analyses
Replication	All attempts at replication were successful (n=3 or more of independent experiments)
Randomization	Randomization was not relevant on experimental designs. I.e. experimental controls were included in all experiments and data analysis was objective.
Blinding	Quantifications of data from all initial experiments were subjected to blinded analysis. Further experiments were not subjective.

Reporting for specific materials, systems and methods

We require information from authors about some types of materials, experimental systems and methods used in many studies. Here, indicate whether each material, system or method listed is relevant to your study. If you are not sure if a list item applies to your research, read the appropriate section before selecting a response.

Materials & experimental systems

n/a	Involved in the study	
☐	☒	Antibodies
☐	☒	Eukaryotic cell lines
☒	☐	Palaeontology and archaeology
☐	☒	Animals and other organisms
☐	☒	Human research participants
☒	☐	Clinical data
☒	☐	Dual use research of concern

Methods

n/a	Involved in the study	
☒	☐	ChIP-seq
☒	☐	Flow cytometry
☐	☒	MRI-based neuroimaging

Antibodies

Antibodies used	All information on primary and secondary antibodies is provided in the manuscript (please see Supplemental Tables 2 and 3)
Validation	All information on validation of primary antibodies is provided by the manufactures (please see Supplier and catalog number information in Supplemental Table 2). Further, the vast majority of primary antibodies were validated by the authors in previous published articles. Validation provided by manufactures:<br>Acetylated α-tubulin: https://www.sigmaaldrich.com/catalog/product/sigma/t9026?lang=en&region=DK&gclid=Cj0KCQjwnqH7BRDdARIsACTSAduFd9Zlcg_tBHqp5W_LMlccQdtoK7ehqEgSrUis80TJLBRFSC0LtDQaAl06EALw_wcB<br>ARL13B: https://www.ptglab.com/products/ARL13B-Antibody-17711-1-AP.htm?

gclid=Cj0KCQjwnqH7BRDdARIsACTSAdvU3PezbLlvzzvfcThP0lWn5JoZNPIzLN5VCu7ga5p9dUT89zF9djQaApyNEALw_wcB
α-tubulin: https://www.sigmaaldrich.com/catalog/product/sigma/t5168?lang=en&region=DK&gclid=Cj0KCQjwnqH7BRDdARIsACTSAdtq-MXNUjQwFhfnKy4uSrmbO-9xSrZCa-8LxBPeqA17HiYNSX6MshgaAv1TEALw_wcB
βIII-tubulin: https://www.abcam.com/beta-iii-tubulin-antibody-neuronal-marker-ab18207.html
BrdU: https://www.citeab.com/antibodies/88841-ma1-82718-brdu-monoclonal-antibody-bu1-75-icr1
GFP: https://www.scbt.com/p/gfp-antibody-fl
GFAP: https://www.citeab.com/antibodies/2452274-z0334-glial-fibrillary-acidic-protein-gfap
MAP2 (rabbit): https://www.abcam.com/map2-antibody-neuronal-marker-ab32454.html
MAP2 (chicken): https://www.abcam.com/map2-antibody-ab5392.html
DCTN1: https://www.bdbiosciences.com/eu/applications/research/stem-cell-research/ectoderm-markers/human/purified-mouse-anti-p150-glued-1p150glued/p/610474
NOL6 (ThermoFisher): https://www.thermofisher.com/antibody/product/NOL6-Antibody-Polyclonal/PA5-30807
NOL6 (Sigma-Aldrich): https://www.sigmaaldrich.com/catalog/product/sigma/hpa055891?lang=en&region=DK
NOL12: https://www.abcam.com/nol12-antibody-ab111704.html
PAX6: https://www.abcam.com/pax6-antibody-ab5790.html
Pericentrin-2: https://www.citeab.com/antibodies/822236-sc-28145-pericentrin-2-c-16
Phospho-RB: https://www.cellsignal.com/products/primary-antibodies/phospho-rb-ser807-811-antibody/9308?Ntk=Products&Ntt=9308
Phospho-CDK1 (Thr161): https://www.cellsignal.com/products/primary-antibodies/phospho-cdc2-thr161-antibody/9114?Ntk=Products&Ntt=9114
RRP7A (Abcam): https://www.abcam.com/rrp7a-antibody-epr14412-c-terminal-ab185225.html
RRP7A (Sigma): https://www.sigmaaldrich.com/catalog/product/sigma/hpa001586?lang=en&region=DK
RRP7A (Santa Cruz): https://www.scbt.com/p/rrp7a-antibody-b-4
SOX2: https://www.rndsystems.com/products/human-mouse-rat-sox2-antibody-245610_mab2018
Vimentin (ThermoFisher): https://www.thermofisher.com/antibody/product/Vimentin-Antibody-Polyclonal/PA1-16759
Vimentin (Dako): https://www.agilent.com/en/product/immunohistochemistry/antibodies-controls/primary-antibodies/vimentin-(concentrate)-76509

Eukaryotic cell lines

Policy information about cell lines

Cell line source(s)	Human dermal fibroblasts (HDFs): skin punch biopsies from 1) affected individuals homozygous for RRP7A, 2) MCPH patients homozygous for a WDR62 mutation, and 3) healthy control individuals. hTERT RPE-1 (RPE-1; ATCC; cat# CRL-4000), HEK293T (ATCC; cat# CRL-3216), P19.CL6 cells: lab stock (originally obtained from Riken Cell Bank, Ibaraki, Japan) .
Authentication	All cell lines were continuously validated by morphology, growth kinetics and/or differentiation status. Mutant/patient cell lines (HDFs and P19.CL6 cells) were continuously validated by western blotting and qRT-PCR analyses.
Mycoplasma contamination	All cell lines were contiguously tested negative for mycoplasma
Commonly misidentified lines (See ICLAC register)	no such included

Animals and other organisms

Policy information about studies involving animals; ARRIVE guidelines recommended for reporting animal research

Laboratory animals	The rrp7a mutant line (sa11429), acquired from ZIRC, were used as adults after being crossed to AB/TL line of 1-2 years old male and female adult zebrafish. All analyses were performed on F5-F7 larvae, generated by crosses of heterozygous 1-2 years old male and female adult zebrafish.
Wild animals	The study did not involve wild animals.
Field-collected samples	The study did not involve samples collected from the field.
Ethics oversight	All experiments were conducted according to the guidelines of the Danish Animal Experiments Inspectorate (License number: 2017-15-0201-01279).

Note that full information on the approval of the study protocol must also be provided in the manuscript.

Human research participants

Policy information about studies involving human research participants

Population characteristics	The five generation family with MCPH originates from the rural area in the Rahim Yar Khan district, in Punjab province of Pakistan. Clinical characteristics of affected family members (two males and six females) are shown in Supplementary Table I. The affected members had head circumference between 41 and 47 cm [-6 to -8 SD below population mean]. Age of affected family members were from 8-30 y.o. Tissue sections from a 19 wpc midgestation foetus was used for immunohistochemical analysis.

Recruitment	Recruitment, examination of patients and construction of family pedigree was performed by experienced clinical geneticists, researchers and postgraduate students. All clinical diagnoses were performed by medical doctors, thus no self-selection bias or other bias are likely to impact results. Informed consent was obtained from all participating individuals or their parents for the collection of blood and/or skin biopsy samples, genetic analyses, and publication of photos and genetic information.
Ethics oversight	The study followed the declaration of Helsinki and was approved by the Institutional Research Ethics Committee, National Institute for Biotechnology and Genetic Engineering, Faisalabad, Pakistan. Human fetal material was obtained in Denmark from legal abortions. Written and oral informed consent was given according to the Helsinki declaration II, approved by the Research Ethics Committee of the Capital Region (KF–V.100.1735/90).

Note that full information on the approval of the study protocol must also be provided in the manuscript.

Magnetic resonance imaging

Experimental design

Design type	Clinical MRI was performed to confirm MCPH phenotype in a single patient
Design specifications	MRI Brain obtained in Axial, Coronal and Sagittal views
Behavioral performance measures	Behavioural performance and functional MRI was not measured.

Acquisition

Imaging type(s)	Transversal Magnetic Resonance Imaging
Field strength	1.5
Sequence & imaging parameters	Multiplanar Multisquential (T1W; T2W Flair and Diffusion Images). Acquisition Time: 1:38 and 0:42. Protocol Name: t2_blade_tra and t2_tse_sag_p2_320. Resolution Matrix: 256x35/320x240. Number of Slices: 19 unary, no units. FoV Read: 230 millimeter. FoV Phase: 100 percent. Repetition Time: 4000-4500 milliseconds. Echo Time: 99 milliseconds. Flip Angle: 150 degree. Protocol Name: t2_blade_tra.
Area of acquisition	MRI Brain obtained in Axial, Coronal and Sagittal views,<br>No.of Slices: 19 unary, no units, Slice thickness (5 millimeter)

Diffusion MRI ☐ Used ☒ Not used

Preprocessing

Preprocessing software	syngo MR B17
Normalization	Not relevant (we did not perform functional magnetic resonance imaging analyses).
Normalization template	Not relevant (we did not perform functional magnetic resonance imaging analyses).
Noise and artifact removal	Not relevant (we did not perform functional magnetic resonance imaging analyses).
Volume censoring	Not relevant (we did not perform functional magnetic resonance imaging analyses).

Statistical modeling & inference

Model type and settings	Not relevant. We did not perform functional magnetic resonance imaging analyses, thus no statistical modeling was performed.
Effect(s) tested	Not relevant. We did not perform functional magnetic resonance imaging analyses, thus no effects were tested.

Specify type of analysis: ☐ Whole brain ☐ ROI-based ☐ Both

Statistic type for inference<br>(See Eklund et al. 2016)	Not relevant (we did not perform functional magnetic resonance imaging analyses).
Correction	Not relevant (we did not perform functional magnetic resonance imaging analyses).

Models & analysis

n/a	Involved in the study
☒	☐ Functional and/or effective connectivity
☒	☐ Graph analysis
☒	☐ Multivariate modeling or predictive analysis
